# Sexualized Deepfake Abuse: Perpetrator and Victim Perspectives on the Motivations and Forms of Non-Consensually Created and Shared Sexualized Deepfake Imagery

**DOI:** 10.1177/08862605251368834

**Published:** 2025-09-09

**Authors:** Asher Flynn, Anastasia Powell, Asia Eaton, Adrian J. Scott

**Affiliations:** 1 Monash University, ARC Centre of Excellence for the Elimination of Violence Against Women, Melbourne, VIC, Australia; 2 RMIT University, Melbourne, VIC, Australia; 3 Florida International University, Miami, USA; 4 Goldsmiths, University of London, London, United Kingdom

**Keywords:** technology- or image-based abuse, victims/survivors, sexual assault, offenders/perpetrators, sexual assault

## Abstract

Advances in digital technologies provide new opportunities for harm, including sexualized deepfake abuse—the non-consensual creation, distribution, or threat to create/distribute an image or video of another person that had been altered in a nude or sexual way. Since 2017, there has been a proliferation of shared open-source technologies to facilitate deepfake creation and dissemination, and a corresponding increase in cases of sexualized deepfake abuse. There is a substantive risk that the increased accessibility of easy-to-use tools, the normalization of non-consensually sexualizing others, and the minimization of harms experienced by those who have their images created and/or shared may impact prevention and response efforts. This article reports on findings from 25 qualitative interviews conducted with perpetrators (*n* = 10) and victims (*n* = 15) of sexualized deepfake abuse in Australia. It provides insights into sexualized deepfake abuse, patterns in perpetration and motivations, and explores theoretical explanations that may shed light on how perpetrators justify and minimize their behavior. Ultimately, the study finds some similarities with other forms of technology-facilitated sexual violence, but identifies a need for responses that recognize the accessibility and ease with which deepfakes can be created, and which capture the diversity of experiences, motivations, and consequences. The article argues that responses should expand beyond criminalization to include cross-national collaborations to regulate deepfake tool availability, searches, and advertisements.

## Introduction

Technology-facilitated sexual violence (TFSV) is a growing problem globally. Advances in digital technologies, machine learning, and artificial intelligence (AI) create new social norms and practices that further blur the divide between public and private life (boyd, 2010), and provide new opportunities for people to engage in harmful sexual behaviors (Henry et al., 2020). The term TFSV is wide-ranging and inclusive of a variety of subtypes of interpersonal sexual violence that capture both online and in-person sexually based harms. For example, using digital technologies to engage in sexual exploitation, sexual and gendered harassment, and image-based sexual abuse (IBSA). TFSV is gendered in both its nature and impacts. Research suggests that women experience TFSV at disproportionately higher rates than men (Brown et al., 2022; Powell & Flynn, 2023; Powell et al., 2022a); that men engage in perpetration at higher rates than women (Henry & Umbach, 2024; Powell & Flynn, 2023; Powell et al., 2022c); and that women experience TFSV concurrently with other forms of violence at higher rates than men (Flynn et al., 2024b; McLachlan & Harris, 2022; Powell & Flynn, 2023). Research has further shown that women report experiencing more significant impacts and harms from TFSV than men (Powell & Flynn, 2023; Powell et al., 2022a).

Communities who experience multiple marginalizations, such as LGBTQI+ individuals, Black and ethnic minority groups, people with disabilities, and culturally and linguistically diverse communities, have also been found to experience TFSV at higher rates, and face additional barriers to seeking support (Flynn et al., 2024b). Much of the research to date has applied feminist theories to explain how TFSV is rooted in gendered power dynamics and societal norms that objectify and exploit women and marginalized communities, thereby normalizing and downplaying the harms of such behaviors. Others have explored TFSV through Kelly’s (1988) continuum of sexual violence, arguing that technology has provided an expansion for men’s typical and aberrant behavior to flourish (McGlynn et al., 2017); or linked TFSV to actor-network theory, in which the behaviors are a combination of human and non-human components that extend “traditional” forms of gendered and sexual violence (Henry et al., 2020; Powell et al., 2018).

An example of the expansion of TFSV is the use of AI to create realistic, but partly or entirely fabricated, non-consensual sexualized imagery. Colloquially known as “deepfakes,” sexualized deepfake abuse is an expanded, but distinct form of IBSA involving the non-consensual creation, distribution, or threat of creation/distribution of an image or video that has been altered in a nude or sexualized way using AI technologies (Flynn et al., 2022b). Sexualized deepfake abuse first came to public attention in 2017, when a Reddit user uploaded sexualized deepfake imagery they had non-consensually created with female celebrities’ faces transposed onto the bodies of pornography actors (Paris & Donovan, 2019). Since then, there has been a proliferation of open-source technologies being shared in online contexts to facilitate sexualized deepfake abuse (Adjer et al., 2019). Among other avenues, this has included online websites and digital platforms that create personalized sexualized deepfake images for users (e.g., a young woman with dark hair, brown eyes, sitting with legs spread on a benchtop); websites and platforms that provide the resources and tools for users to create their imagery in exchange for payment or by posting the images to the platform’s community; and the development of free and pay-to-use nudify apps, in which a user can create a sexualized deepfake using any image by digitally erasing the person’s clothing and replacing it with genitalia (Szyf et al., 2024).

Over the last 12 months, there have been numerous reports of sexualized deepfake abuse, including high-profile celebrities (e.g., Taylor Swift), as well as cases involving schoolgirls and female teachers across Australia, Europe, the United Kingdom, and the United States (Flynn et al., 2024a; Hurtado, 2023; Narvali et al., 2023). While the non-consensual creation and sharing of sexualized imagery has been the focus of substantial research and legal intervention (Flynn & Henry, 2021; Henry et al., 2021; Patel & Roesch, 2022), there remains a dearth of research examining the nature of sexualized deepfake abuse, its impacts on victims, and analysis of perpetrator motivations. What is notably lacking is knowledge of perpetrator experiences of sexualized deepfake abuse (this is true of most TFSV perpetration) and victim experiences of sexualized deepfake abuse.

This article contributes to this pressing knowledge gap, recognizing the vital, lived-experience knowledge that is held by perpetrators and victims of sexualized deepfake abuse. Presenting findings from 25 qualitative interviews conducted with perpetrators (*n* = 10) and victims (*n* = 15) who engaged in or experienced sexualized deepfake abuse, this article responds to two primary questions: (a) What types of sexualized deepfake abuse behaviors are engaged in and experienced, as identified by a cohort of perpetrators and victims of sexualized deepfake abuse; and (b) what are the self-disclosed and perceived motivations of sexualized deepfake abuse perpetration, as identified by a cohort of perpetrators and victims of sexualized deepfake abuse?

In answering these questions, we adapt existing theories of male peer support and homosociality (DeKeseredy, 1988), moral disengagement (Pina et al., 2021), and techniques of neutralization (Sykes & Matza, 1957) to explore how perpetrators minimize and justify their behaviors, mitigating feelings of guilt or responsibility. The findings contribute to improved understandings of the motivations, social norms, and justifications underpinning sexualized deepfake abuse, and provide insight into different forms of sexualized deepfake abuse, including the tools, software, and images used in the creation process. Ultimately, our findings show some similarities between sexualized deepfake abuse and other forms of TFSV, but highlight the need for a broader response that recognizes the accessibility and ease with which images can be created, and that captures the diversity of experiences, motivations, and (lack of) consequences. Our findings further suggest that responses to sexualized deepfake abuse need to expand beyond existing criminal law interventions to include legal regulation of deepfake tool availability, searches, and advertisements on websites and digital platforms. Such research is critical for informing legal, regulatory, and policy approaches to these harmful behaviors, and to develop recommendations for appropriate prevention and responses. To the best of our knowledge, this is the first peer-reviewed research to report on interviews with perpetrators and victims of sexualized deepfake abuse.

## Sexualized Deepfake Abuse: Understanding an Emerging Harm

Much of the research on harmful and non-consensual sexualized image creation and sharing has focused on unedited IBSA (McGlynn et al., 2021; Ruvalcaba & Eaton, 2020). Indeed, almost 10 years of research into the extent and nature of IBSA has demonstrated not only that non-consensual image creation and sharing is increasingly common (Powell et al., 2020a, 2020b, 2022b, 2022c), but that perpetrators and bystanders normalize IBSA behaviors through cultural and cognitive mechanisms that downplay the harms they cause (Flynn et al., 2022b, 2023a). For example, Morales et al. (2024) found that Canadian men endorsed a variety of myths to normalize TFSV, including IBSA, such as “he didn’t mean to” or “she lied.” Other studies have found that perpetrators and bystanders view TFSV as less severe because it occurs in a virtual (vs. in-person) space (Fisico & Harkins, 2021; Worsley & Carter, 2021).

Perpetration research focused on IBSA has found that such behaviors are not always motivated by malicious intent (Eaton et al., 2017; Powell et al., 2022c). Self-reported reasons for perpetrating IBSA among adults in the United States, for example, have ranged from “just sharing the image(s) with my friend for fun” to “for the upvotes”, referring to the social endorsement of IBSA content between peers and in online communities (Eaton et al., 2017). These justifications fail to reflect an awareness of having perpetrated sexual abuse or the harm it causes. This minimization both trivializes sexual violence and reduces empathy for victims.

The relationship between the normalization of sexual violence and victim-blaming is well-documented in psychological and sociological research (Hayes et al., 2013; Suarez & Gadalla, 2010), including studies of attitudes toward IBSA victims (Flynn et al., 2023a). Similar examples of minimization have been presented in relation to sexualized deepfake abuse not being as harmful as other forms of IBSA because the image is fake. Prior to introducing new legislation in 2023, this was the justification provided for not including sexualized deepfake abuse within IBSA laws in England and Wales (Flynn, 2023). Similarly, in a recent study in the United States surveying public perceptions of deepfake content (both sexualized and in other forms), a perceived lack of harm was the most common reason identified for finding the creation of that content acceptable (Brigham et al., 2024). While the study reported that most respondents found the creation of sexualized deepfake images unacceptable, there was a gap between this perception and the relative acceptability of searching for and viewing non-consensual sexualized deepfake images. In exploring potential reasons underpinning this gap, Brigham et al. (2024, p. 6) suggested it was because the harms of sexualized deepfake abuse are “not fully appreciated by many people.” They suggest anonymity in viewing the imagery, combined with a distance between the viewer and the victim, may reduce understandings of sexualized deepfake abuse harms and lead to indifference.

Research into the various forms of sexualized deepfake abuse is very much in its infancy. While literature has examined the legal and policy challenges for regulating and/or responding to sexualized deepfake abuse (Flynn et al., 2022a; Kirchengast, 2020; Lucas, 2022), studies reporting on the extent and/or experiences of these harms are particularly sparse. In a study of sexualized deepfake content circulating on X (Twitter), Maddocks (2020) found that sexualized deepfake abuse disproportionately represented and targeted women, and was a mechanism for silencing women, reflecting broader patterns of gender inequality. Taylor (2023) similarly positions sexualized deepfake abuse as a form of men’s gendered sexual violence against women, arguing that such content overtly intends to sexually humiliate women.

In one of the few quantitative surveys examining victimization and perpetration, Flynn et al. (2022b) found that over 14% of 6,109 respondents across Australia, New Zealand, and the United Kingdom had experienced someone creating, distributing, or threatening to distribute a digitally altered image representing them in a nude or sexualized way, and around 8% self-reported perpetrating such behaviors. Men were significantly more likely than women to report perpetration, as were younger respondents (Flynn et al., 2022b). Other recent quantitative research has focused on attitudes toward the creation of sexualized deepfake imagery, finding that women were significantly more likely than men to support criminalization and to condemn sexualized deepfake pornography generally (Umbach et al., 2024), and that those with higher levels of psychopathy showed more lenient judgments toward the creation of sexualized deepfake pornography and higher proclivity to perpetrate (Fido et al., 2022).

The current article builds on this research, providing unique qualitative insights into perpetrator and victim experiences of sexualized deepfake abuse, including potential motivations and justifications for the behaviors. While a small body of research has revealed some quantitative predictors of sexualized deepfake abuse attitudes and behavior, there is no qualitative research to our knowledge examining perpetrators’ and victims’ subjective understandings of why, and in what contexts, this abuse is perpetrated. The current study, therefore, contributes to theory by helping us understand social norms and narratives around TFSV and sexualized deepfake abuse, which should be targeted in prevention efforts such as deterrence messaging and sex education.

## Theoretically Framing Perpetration

### Male Peer Support

Originally coined by DeKeseredy (1988), and redeveloped by DeKeseredy and Schwartz (1993, 2013, 2016) over several iterations, male peer support theory is used as a basis to explain why some men “situated in a patriarchal rape-supportive culture, have male friends with similar beliefs and values who act to develop and then reinforce beliefs and values that promote the abuse of women” (DeKeseredy & Schwartz, 2016, p. 4). In other words, male peer support allows men to feel normal and justified when committing gendered violence. The theory builds, in part, on the work of Connell (1983, 1987) and Connell and Messerschmidt (2005), whose concept of hegemonic masculinity highlighted the varied norms and mechanisms through which the “ideal” or dominant ways of being a man are reproduced throughout societies and localized communities. As Connell and Messerschmidt (2005) acknowledge, in many societies, hegemonic masculinity is characterized by dominance, physicality, aggression, and violence, and is reinforced through processes of social relations, including peer groups.

Over several decades, male peer support theory has offered unique and localized insights into the processes of peer group relations that both produce and reproduce masculine norms and practices linked to violence against women (DeKeseredy & Schwartz, 1993, 2013, 2016). The types of support provided by male peers may take various forms, including routine activities, such as sports or socializing in bars where abusive attitudes can be reinforced; informational support, where advice and guidance is shared that encourages abusive behaviors; the forming of bonds or attachments with peers who have a history of abusing women; and peer pressure, where men are pressured to conform to group norms that include abusive behaviors (DeKeseredy, 1988). This theory also considers how stressors in relationships, such as challenges to perceived male authority, can lead men to seek support from peers, which may encourage abusive actions (Henry & Flynn, 2019). The theory has been used to understand the social dynamics that contribute to IBSA to help inform prevention and intervention strategies, and to challenge IBSA and sexting education campaigns that minimize or excuse perpetration and instead place blame onto the victim (Zauner, 2021). In this article, we explore whether male peer support can similarly be used to better understand sexualized deepfake abuse perpetration.

### Techniques of Neutralization and Moral Disengagement

Almost 70 years ago, Sykes and Matza (1957) identified five techniques of neutralization that individuals use to justify or rationalize their deviant behavior to mitigate feelings of guilt and maintain a positive self-image. These include the following: denying responsibility (claiming actions were due to external forces beyond their control), denying injury (claiming they did not cause any real harm), denying the victim (minimizing the harms to the victim or placing blame on them), condemning the condemners (shifting the focus to those who disapprove of their behavior), and appealing to higher loyalties (claiming they were serving a higher purpose). These techniques of neutralization apply when perpetrators acknowledge that their actions may violate societal norms, yet still justify engaging in these acts, thereby neutralizing the controls that would normally govern their behavior. This theory has been used to explain perpetration of a range of cybercrimes, including hacking (Bossler et al., 2011; Chua & Holt, 2016) and piracy (Brown, 2016; Holt & Morris, 2009), but is less frequently applied to TFSV (see van Baak et al., 2022 for an exception on cyber harassment).

Building on these techniques, Pina et al. (2021) have explored whether moral disengagement theory can potentially explain how perpetrators of IBSA engage in harmful behaviors without feeling personal guilt or responsibility. In their study, Pina et al. (2021) found that perpetrators used various cognitive mechanisms to justify their actions and mitigate feelings of guilt, including denying responsibility, minimizing harm, and blaming the victim. In this article, we consider whether similar techniques have been applied by perpetrators of sexualized deepfake abuse.

## Methodology

In this study, we define perpetrators as those who have engaged in sexualized deepfake abuse (i.e., non-consensually created, shared, threatened to create, or threatened to share, sexualized deepfake images of another person), and victims as those who have experienced sexualized deepfake abuse (i.e., had someone else non-consensually create, share, threaten to create, or threaten to share, sexualized deepfake images of them). Australia was selected as the focus country due to its criminalization of IBSA and sexualized deepfake abuse, and other globally advanced responses, such as empowering the eSafety Commissioner to compel digital platforms to remove non-consensually created sexualized deepfake imagery, or face legal and financial penalties (Flynn, 2023).

In June 2024, 25 qualitative online interviews were conducted with adult perpetrators (*n* = 10) and victims (*n* = 15) of sexualized deepfake abuse across Australia using the Zoom platform. The primary aim of the interviews was to gain deep, context-specific insights into perpetrator and victim experiences of sexualized deepfake abuse. Three researchers (two female professors with significant experience in trauma-informed and qualitative interview practice; one male research assistant with experience working in behavior change and perpetrator programs) undertook the interviews, and, where possible, participants were interviewed by someone who identified as the same gender. This decision was made based on previous research the team has undertaken, in which male perpetrators provided more details of their involvement in engaging in interpersonal harm when speaking with male interviewers. Ethics approval was received from the Monash University Human Research Ethics Committee.

### Sample and Participants

A purposive sampling approach was used in which participants were selected based on specific experiences (a perpetrator or victim of sexualized deepfake abuse). Due to the inherent difficulties of recruiting interview participants in TFSV research (Boxall et al., 2023; Flynn et al., 2024b), and to avoid some of the privacy and security limitations of recruitment via social media advertising (Archard & O’Reilly, 2022; Oudat & Bakas, 2023), a recruitment company was used. The company sent a series of invitations to participate in the project to people on their database who had consented to be contacted for research purposes. It is unknown how many invitations were sent, but a total of 33 prospective participants self-reported matching the eligibility requirements (aged 18+ years, engaged in or experienced sexualized deepfake abuse) and, after further clarification of eligibility confirmed by the lead author, were invited to an interview. A total of 25 participants consented to participate and were interviewed. All were provided with a small honorarium to recognize their involvement.

The target sample size was initially identified based on comparative peer-reviewed research with similar hard-to-reach target groups of TFSV perpetrators (Flowers et al., 2020; Flynn et al., 2024b) and studies with victims (Flynn et al,, 2024b; St. Vil et al., 2018). During the interviewing process, we reached data saturation against the research questions for our non-representative purposeful sample (Guest et al., 2006); however, we completed the remaining scheduled interviews to ensure a range of perspectives relevant to the research questions were included (Hennink et al., 2017).

We attempted to recruit across a range of demographics, including gender, age, ethnicity, and sexuality. However, there were clear gendered patterns in both cohorts (more men identifying as perpetrators and women identifying as victims), as well as a leaning toward heterosexuality. Of the 10 perpetrators, 8 were identified as men and 2 as women. Their ages ranged from 22 to 53 years, with an average age of 36.9 years. The majority identified as heterosexual (*n* = 6), followed equally by same sex attracted (*n* = 2) and bisexual (*n* = 2). Six perpetrator participants described their racial identity as White, and the remainder identified as Asian (*n* = 4). Of the 15 victims, 11 were identified as women and 4 as men. Ages ranged from 18 to 49 years, with the average being 33.5 years. Most identified as heterosexual (*n* = 10), followed by same sex attracted (*n* = 3) and bisexual (*n* = 2). Eight victim participants described their racial identity as White. Other racial identities included Asian (*n* = 3), Middle Eastern (*n* = 2), and African (*n* = 2).

## Materials and Procedure

The interviews were designed to investigate sexualized deepfake abuse, including perpetrator and victim characteristics; how images were sourced; what tools or software were used; and when the image was shared, where, and how it was disseminated. We also sought to understand motivations and perceived harms, as well as any responses to the behavior, supports sought, and participants’ knowledge of IBSA and/or sexualized deepfake abuse laws.

The perpetrator questions were divided into three sections: *the story of deepfake abuse perpetrated* (e.g., “can you tell me about any of these four behaviors you have engaged in?”; “where did you source the original images or videos used?” “what tools/software did you use?”; “who was the subject of the image?”); *motivations and harms* (e.g., “what do you think motivated you to do this?”; “how do you pick who to create/distribute the image of?”; “did you consider your behavior to be potentially harmful at the time?”); and *responses* (e.g., “did you tell anyone you had done this?”; “did you know that the behavior could have legal ramifications, such as being charged by police?”; “did any authorities become involved?”).

The victim questions were similarly divided into three sections and followed the same pattern, but the focus was changed to their experience and perceptions of the motivations: *the story of deepfake abuse experienced* (e.g., “can you tell me about any of these four behaviors you have experienced?”; “how did you find out about the image and/or video?”; “do you know who the perpetrator was?”); *motivations and harms* (e.g., “why do you think the perpetrator created/shared the image?”; “what impacts did this have on you?”); and *responses* (e.g., “did you tell anyone about this experience?”; “did you seek any support?”; “did you report the behavior to anyone?”). In this article, we report on the story of the deepfake abuse and perpetrator motivations (actual and perceived).

The interviews lasted between 30 minutes and 1 hour, with an average duration of 48 minutes. Given that the discussion involved the misuse of imagery, participants were only given the opportunity to be interviewed via Zoom audio or Zoom audio and chat. All chose Zoom audio. The interviews were recorded and transcribed using Zoom auto-transcription. The lead author reviewed the transcripts for errors and de-identified them.

### Data Analysis

The de-identified transcripts were input into Dovetail, a qualitative data analysis platform that allows research teams to code and analyze data thematically and in real-time across devices. A reflexive thematic analysis was undertaken, following five key steps (Byrne, 2022; Clarke & Braun, 2021). This began with the lead author developing a coding frame that reflected the research questions to guide the analysis and then reviewing the transcripts, identifying initial impressions, codes, and ideas. Next, initial codes were generated using a combination of data-driven (inductive) and theory-driven (deductive) analysis, informed by the interview questions (e.g., “what do you think motivated you to do this”; “why do you think the perpetrator created/shared the image”; “did you tell anyone you had done this”) and relevant literature (e.g., DeKeseredy, 1988). Example codes included the following: *sexualized deepfake abuse behaviors* (“create,” “share,” “threat to share,” “threat to create”) and *harms* (“emotional,” “social,” “physical,” “financial”). This was shared and reviewed by the second author to validate the approach. Broader patterns and themes were then identified, and data relevant to each theme were collected. Example themes are as follows: “perpetrator motivations,” “normalization/male peer support,” and “victim impacts.” Quotes were then selected from the transcripts to help illustrate each of the themes and incorporated into the article. Pseudonym names are used throughout this article alongside a perpetrator (P) or victim (V) identifier (e.g., Eugene_P, Emilia_V).

## Results

### Sexualized Deepfake Abuse Experiences

In the study, we defined four forms of sexualized deepfake abuse, which participants were invited to share their experiences of *create*, *share*, *threat to create*, and *threat to share*. For all categories, AI, digital tools, or software (such as an app) had to be used (or threatened to be used) to create sexualized imagery of another person that they knew (e.g., friend) or did not know (e.g., celebrity), without that person’s knowledge and/or approval. All 10 perpetrators reported having engaged in both the creation and sharing of images; 1 perpetrator reported having created, shared, and threatened to share images. Deon_P, for example, described his behavior as follows:

I have been involved in making them . . . faking sexual images of her. . . . They were real sexual images, but we faked them in some cases or made them look worse than they were . . . like sticking butter up her pussy.

When an image was shared, this occurred in a variety of ways. Most commonly, the images were sent by direct message to the victim, the victim’s friends/family members, or the perpetrator’s friends via messaging services such as “private chat groups, so WhatsApp and Messenger” (Idris_P). In describing sharing sexualized deepfake images of his ex-partner, Jess_P revealed:

It was posted in WhatsApp. I was tempted to use some of those other [pornography] websites to ruin her professional career a bit . . . but I didn’t do that. I just simply used the personal WhatsApp groups. And some of these groups had up to 50 people.

Other perpetrator participants described uploading and sharing the images on social media platforms or dating apps, including “Grinder, . . . Hinge, and one called Hornet” (Eugene_P). In one interview, where the justification offered for the behavior was “humor,” Luca_P described having the images “printed onto t-shirts and kind of distributed that way as well.”

The tools used to create the images ranged from “literally Googled it . . . found a website and just put the picture in and it kind of generates itself” (Luca_P), to nudify apps where you “build and nudify a photo. You can copy and paste a normal photo, and it makes it a nude one” (Eugene_P), right through to “an app for the regular manipulation of photos and faces, but . . . for videos, which are a little bit more comprehensive, [friend] has a subscription to a lot of them and we use those” (Jesse_P). These methods suggest a degree of simplicity and ease in locating the tools required to make an image, and the lack of skills or training needed to perpetrate sexualized deepfake abuse.

Regarding victimization, nine victims reported experiencing creation, eight reported experiencing sharing, eight reported experiencing the threat to create, and seven reported experiencing the threat to share. This suggests a common pattern of both poly-victimization and poly-perpetration among our sample. Victim experiences highlighted the diversity of ways in which the sexualized deepfake image was created and was often then shared:

There were photos of me that were taken and then digitally altered. . . . It was just like you know, a sexual image, a naked image, and it got shown around. . . . It got air dropped (Emilia_V).

It was my head on pornographic pictures, on their bodies and things like that. They did look very much like me (Stella_V).

Most victim participants were made aware of the creation and sharing of the image by the perpetrator or by family or friends:

He [the perpetrator] sent it to me directly, and then it was also sent to people that I know (Kim_V).

I was at my job and then I was getting messages from people saying that there was an account that was created in my name using my pictures, and there was a link saying that I had created an OnlyFans account. . . . It was like, my face on someone else’s body. So even the things that they were posting, it was like a mixture of my images and then my face on another woman’s naked body (Taylor_V).

Other victim participants reflected on discovering that a sexualized deepfake image had been created and shared of them, for example, on “Reddit” (Zainab_V) and “Facebook” (Kim_V), when another person brought it to their attention:

I received an email from a person. I have no idea to this date who they are, where they’re from, or what the connection was. And within that email was a link to a website on which an image of me had been uploaded and shared (Poppy_V).

In some instances, the images were shared on pornographic websites, often in addition to being shared in private messaging groups, as Ashanti_V described, “those kinds of porn sites where it’s free to share those things. And then, like Snapchat, Instagram, Telegram.”

For those victim participants who experienced threats to create or share imagery, this was always directly from the perpetrator—whether that person was known (i.e., a partner or friend) or unknown (i.e., an unknown number or email) to them personally:

It was a friend of mine. . . . He was threatening to make disrespectful and naked photos of me, and then send them to others. He said, “I can do this, and you know, I can edit a photo, and no one would know [it’s fake]. I can say that you sent that to me, and I can share that with others” (Zara_V).

After we were videoing on Skype for a bit, I could just tell something was up. . . . So, after ending the Skype call and blocking the person that I was speaking with, I did get a text message on WhatsApp and whoever was behind it, ended up finding my Facebook page and there was an image on it. . . . It was a shirtless photo, so I suppose it was pretty easy for them to make some sort of sexual picture out of it. And they did end up placing my body onto—it’s as if I’d sent them a naked photo. . . . And they did end up sending that photo to a friend of mine (Lincoln_V).

Collectively, these findings demonstrate the variety of ways in which sexualized deepfake imagery can be shared, ranging from within the perpetrator and victim’s social or family networks, through to large public domains in which those viewing the images may be known and unknown to the perpetrator (and victim). This suggests a range of motivations may be underpinning the behaviors, beyond a desire to harm the victim.

### Motivations

Four key motivations emerged from the interview data: money (sextortion), curiosity, causing harm, and peer reinforcement. Of these, monetary gain was identified only by victim participants, curiosity was identified only by perpetrator participants, while themes around causing harm and peer reinforcement were acknowledged by both participant groups.

#### Money (Sextortion)

In four of the victim interviews, participants described instances akin to sextortion, where they had experienced sexualized deepfake abuse after meeting someone online. In these examples, the victim had interacted in a non-sexual manner with the person and was later threatened that another party would non-consensually share sexualized deepfake images of them, unless they paid money. Zainab_V experienced this after she had a video call with a woman she met on Facebook. The content of the call was “friendly” with no sexual element, but after the call ended, she received sexualized deepfake images and videos of herself via Facebook Messenger, alongside threats to share these with her Facebook friends if she did not pay money. In another interview, Lincoln_V described having a video call with someone who attempted to get him to engage in sexual activity (taking clothes off, touching genitalia), which he did not do. After ending the call, he had pictures lifted from his social media account, which were then altered into sexualized deepfake images. He was threatened that these would be shared with his friends if he did not pay money. He refused, and the images were then shared with at least one Facebook friend (to his knowledge).

#### Curiosity

Curiosity in the process of creating the fake image and the ease with which images could be created was a driving factor identified by some perpetrator participants. As Nova_P explains:

You just want to see what’s possible. And you just like get an idea in your head, and you want to see what that’s like. And so, you create it. And then you have a little God like buzz of seeing that you’re capable of creating something like that.

It was common for perpetrator participants to reflect on how easy it was to create the image. Deon_P observed, “the online AI tools . . . we just throw the pictures in and just sort of use it to make it. . . . It’s easy.” Malik_P similarly claimed, “It was quite a simple task. It wasn’t very complex at all. And I was actually really surprised at how easy it was to sort of do that. . . . I actually found it quite scary.”

Curiosity was a particularly common theme among middle-aged perpetrators (35+ years) who reflected on the innovations in technology fueling their initial interest in exploring deepfake tools:

Curiosity mixed with like creativity. . . . The lack of that technology growing up, not being surrounded by those types of tools when I was younger, makes it more interesting and . . . more tempting to do now, because even now, if you create an image like that, it is still quite profound that’s even possible with technology (Osmar_P).

Even for participants who identified other motivations as their primary goal, curiosity was still recognized as a contributing factor to engaging in the behaviors. As Deon_P explained, “I’m quite interested in AI. I mean, that’s part of why I got involved, because I use a lot of AI tools in my work. So, I’ve seen how powerful some of them can be.”

#### Causing Harm

Where causing harm was identified (often through what participants described as “seeking revenge” for a perceived injustice), it was only in the context of intimate partner or ex-partner relationships, or where a romantic interest was not reciprocated. For example, Eugene_P had created a fake profile on three dating apps impersonating his ex-partner after their relationship ended. He stole photos from his ex-partner’s Facebook page (acknowledging he would not use images “that could be traced to me”) and used AI software to alter these into sexualized deepfake images. He housed the fake imagery in private folders on various dating apps, allowing access to anyone who interacted with him. In describing the driving motivation for his behavior, Eugene_P explained:

He broke up with me, and he cheated on me. . . . So, he deserved this. . . . I was very angry at that time. I just wanted a little bit of revenge. I wouldn’t do this to just anyone, but because this guy, he treated me like this, that should be something that he should pay for.

In another example of perceived wrongdoing in the aftermath of a break-up, Jesse_P described how he had altered and shared sexualized deepfake images of a female employee he had been intimately involved with after the relationship ended. He circulated the edited images through WhatsApp groups associated with their place of employment, and one group that included the victim’s adult daughter. In reflecting on his behavior, Jesse_P claimed it was out of character, but he was fully aware of the harm he might cause and felt “out of control” with anger. He explained:

I’m normally not a person who would hurt anybody, and I was just really surprised in retrospect. You know, the extent I went through, and all the evil thoughts I had to get back at this person, because normally I would not behave in that way. . . . It brought out a lot of nastiness, which I never experienced before. . . . It was a reaction for what she did, and that [the break-up] hurt significantly.

While neither participant specifically acknowledged their behavior as a form of intimate partner violence or coercive control, Stella_V, who experienced sexualized deepfake abuse from her ex-partner, identified the behavior as an extension of his abuse. She explained, “it was definitely designed to humiliate me with my family. . . . I was leaving. He wanted to humiliate me. He was not a really great person. It was about control.”

This sense of entitlement over an ex-partner also emerged in the examples provided by participants who had experienced sexualized deepfake abuse by a male acquaintance (in one instance, a friend, in the other, a colleague) who expressed a romantic interest that was not reciprocated. As Ashanti_V reflected, “the motive is just to humiliate right? Just to like, have one over you. It’s . . . a misogynist motive. Their motivation is to make women feel less than and dehumanized.” Zara_V similarly reflected:

He probably just wanted to hurt me, because he felt hurt about what I had done, even though it wasn’t intentional, and I was, you know, very respectful [in not reciprocating his romantic interest] and I think it’s just about taking revenge and feeling powerful, like I can do this, and there’s nothing you can do about it. And a feeling of making that other person feel helpless.

Research has consistently shown that women are most at risk of violence when a relationship ends, or in the context of a sexual or dating advance, where the man experiences rejection and humiliation (Capaldi et al., 2012; Morgan & Chadwick, 2009; Woerner et al., 2018). While not removing any responsibility from those engaging in these behaviors, there is a significant danger that the accessibility of AI technologies that create sexualized deepfake images will become a more common form of intimate (ex-) partner and dating violence, unless substantial education and prevention responses are put in place.

#### Peer Reinforcement

Peer reinforcement was a common theme emerging in participant explanations for sexualized deepfake abuse perpetration. This was often identified in the context of “humorous” humiliation, for example, when the perpetration was engaged in by a man against a male friend, and the perpetrator then received positive reinforcement from his male peer group. Luca_P, for example, described creating:

A few digital images and videos of mates for a couple of bucks’ parties and things like that, just a bit of a stitch up for them. So, videos and pictures of them performing [sexual] acts, or involved in [sexual] acts . . . with other men. . . . It was humorous. It was funny.

Idris_P similarly described engaging in sexualized deepfake abuse against his male friend because he “was getting teased a little bit for acting a bit gay, so that was part of the motivation for making the imagery.” When asked to expand on this, Idris_P said, “he was displaying characteristics that one may associate with someone who is gay, so . . . it’s building on that, capitalizing on that . . . so people think it’s funny.” This motivation expanded beyond same sex victimization and was also identified by victims of the abuse. In describing her experience of sexualized deepfake abuse perpetrated by a male classmate, Ashanti_V claimed:

They just want to one up and get a laugh out of their friends as well. There is an aspect of wanting to socially, you know, please other men by engaging in this kind of behavior and having a laugh about it.

Further evidence of sexualized deepfake abuse being linked to peer reinforcement was the high number of perpetrators who admitted partaking in other forms of TFSV, with 7 of the 10 perpetrators acknowledging they had engaged in IBSA behaviors involving non-faked imagery. Some participants who identified engaging in both sexualized deepfake abuse and IBSA claimed there was a difference in the motivation and reward between sharing “real” sexualized images of a partner and sexualized deepfake images. Louis_P, for example, reflected on non-consensually sharing “real” images among his male peer group:

It was more conquest than anything or a bit of oh, yeah, look who I’ve picked up on the weekend. . . . It was more like gloating behavior . . . you know, look what I did this weekend, boys, and look, you’ve seen her at Uni[versity], now look at her without clothes on. . . . It’s a trophy. Everyone’s, giving pats, high fives and things like that.

When describing his motivations for engaging in sexualized deepfake abuse, Louis_P was still seeking affirmation and reinforcement from his peers, but he claimed this focused on demonstrating his creativity or skillset, not on building his sexual status. He explained:

They say, “wow, that’s awesome”. You know, like they were in awe. “How’d you do that?” It was more self-pride in the image more than anything, like we weren’t, you know, anti-women. . . . No one said, “don’t send that”. It was more like, it was accepted behavior.

This form of peer reinforcement reflects findings from Hall et al.’s (2021) study on perpetrators and upskirting, whereby homosocial bonding and peer status were evaluated through recognition of the “craftsmanship” of the image. In Hall et al.’s (2021) study, this peer reinforcement was described as “polite misogyny,” in which “the male gaze apprises and appreciates the female body, as if it were an object. . . . The appraiser is imbued with a sense of skillful, knowledgeable judgment; the ‘upskirter’ becomes a skilled craftsman taking pride in his pursuit, rather than a perverted peeping tom” (p. 545). In this regard, the reactions of Louis_P’s male peer support group can be framed in a similar way: viewing the woman in the deepfake image as merely an object, and praising Louis_P for the creativity of the image, thereby ignoring or downplaying the abuse or the reinforcement of misogynistic behaviors through the creation and sharing of a sexualized deepfake image.

Luca_P reflected on his experiences engaging in IBSA and sexualized deepfake abuse as both creating a sense of “peer pressure” for him to continue to engage in the behaviors, and as “encouragement” for the behaviors, giving him more status among his male peers. In the context of IBSA, where he acknowledged “sharing around [naked] pictures I’ve received and videos of girls and things that you forward on, share with group chats or friendship groups,” Luca_P described the motivation as being “getting sort of attention or praise, wanting to perform for the group, or be accepted, you know, be one of the boys.” When reflecting on his sexualized deepfake abuse experiences—in this instance, creating images of male friends in sexual scenarios with other men—while initially describing the motivation as being to “tease and rib on his friends,” the positive peer reinforcement he received from his group of male peers, largely reflective of the status he received from sharing “real” sexualized images, also clearly motivated the behavior:

I always enjoy having a laugh and . . . [being] center of attention and things like that. So, there’s always that pressure on me to perform or to play out to the crowds and things. So, it was, for me, it was just another avenue of that [status building].

### Neutralization of Behaviors

The disconnect between participants’ understanding of sexualized deepfake abuse as harmful and acknowledging their behavior as harmful was another clear theme emerging in the interviews. Participants would commonly minimize or deny the injury or harm to the victim in their experience, even when acknowledging that the behavior could be problematic in other contexts:

Now people were like, thought it was quite fantastic. Honestly, like, you know, because of the nature of this, I can understand some people who could have something made about them and it could be quite compromising . . . but the nature of this [situation], it was fine. It was quite funny (Malik_P).

This neutralization of behaviors was expressed by perpetrators and victims, suggesting that minimization was both a justification technique and a coping mechanism (potentially to reduce stress and maintain control):

He thought it would have been funny, but clearly it got taken way too far, and it wasn’t funny at all, like I don’t think it was. I don’t think he was trying to destroy me, or, like, you know, do something bad to me, I think it was just, you know, a joke, a very stupid joke that went too far (Emilia_V).

This approach was expanded on by Idris_P in relation to intent:

The harmful situation is where it’s being done maliciously and being done in such a way to actually make it seem like it’s a real image. And you know, someone in a compromising position and then sharing it with the community to do damage to their reputation. And the non-harmful ways are more when it’s clearly done as a joke.

Minimizing the harm and denying injury to justify the behavior was also evident in the way participants described their acts. Jesse_P, for example, described his behavior (in this case, creating and sharing quite graphic and professionally sourced sexualized deepfake imagery of an ex-partner) as “art,” and as something that gained him a lot of attention: “When you put up these types of compromising videos and art you get a lot of reaction, some very surprised, some you know, a lot of lengthy conversations.” Even in this description, using the term “art” highlights how Jesse_P sought to deny the injury caused to the victim and engage in what Sykes and Matza (1957) refer to as an appeal to higher loyalties.

In other contexts, the harms of the behavior were minimized through various forms of victim-blaming or denying the victim. In describing using the images of the ex-girlfriend of a member of his male online community, Deon_P justified his engagement in sexualized deepfake abuse behaviors as “pretty menial . . . pretty immature, but not harmful.” He described how up to 20 people from this online community were involved in editing and circulating images that they had accessed from an OnlyFans account, without the woman’s consent. While he noted that a couple of people raised concerns, the consensus was that since she had put the images online herself, it was “fair game” to edit and share them within their private group. He explained:

I kind of just subscribed to the mentality that if you don’t want something out there, you don’t put it out there. As far as like making the edits is considered, yeah, obviously it’s not, she wasn’t involved directly, but I guess it was, she was definitely more of a catalyst in this case. We didn’t really see her side of it, so to speak and . . . we didn’t really care. . . . I personally see the cases of where it’s non-consensual stuff, like editing somebody’s OnlyFans, I don’t see it as a problem.

This form of victim-blaming or denial of injury is not unusual in IBSA research, particularly in cases involving sex workers or where the person in the image consensually sent the image to another person, who then shares it further without their consent (Flynn et al., 2023a, 2025; Scott & Gavin, 2018).

The absence of consequences for engaging in sexualized deepfake abuse also contributed to a reduced perception of its harm. In our study, no perpetrator reported having been legally sanctioned for their behavior, with only one saying the victim (his ex-partner) had threatened to go to the police, but did not. Three victims attempted to report to police, but no action was taken, and unhelpful—harm minimizing—advice was provided:

I reported it twice to the police with the advice of changing my phone number (Kim_V).

I called the police, and they were like, just delete the photo . . . They didn’t think it was a big enough deal (Talia_V).

Others described feeling “too embarrassed . . . the last thing I would have ever done was get police involved, like I just wanted to not even think about it, not tell anyone” (Zara_V), or thinking it was not serious enough or the police would not take it seriously:

I didn’t think it was serious enough to be reported. . . . I thought that if something else happened, and it kind of escalated and went the next step, then that’s when I would. I felt they [the police] wouldn’t care if I did (Page_V).

Other ways the harms were minimized materialized from victims themselves, who compared their experiences with victims of physical forms of abuse:

I was never physically harmed. I was never, you know, none of that. It was mainly just over social media, like just being held hostage essentially with these photos (Ashanti_V).

That’s what makes it worse. I think that the fact that they haven’t ever harmed me physically makes you question yourself like, well, is this person really bad because they’re not bad in other senses? I mean, it’s not like they’re bashing you (Talia_V).

Akin to other forms of non-physical sexual violence or image-based harassment (and indeed, victim experiences of reporting sexual violence), these findings suggest there are complexities in recognizing the harms or reporting sexualized deepfake abuse; the impacts of which may reduce the consequences of, and deterrence from, perpetration.

## Discussion

The primary aim of this study was to gain deep, context-specific insights into perpetrator and victim experiences of sexualized deepfake abuse, and to better understand: (a) the types of sexualized deepfake abuse behaviors engaged in and experienced; and (b) the self-disclosed and perceived motivations of sexualized deepfake abuse perpetration. Within our sample, all 10 perpetrators reported having engaged in both the creation and sharing of images, while 9 victims reported experiencing creation, 8 reported experiencing sharing, 8 reported experiencing the threat to create, and 7 reported experiencing the threat to share. This highlights the diversity of ways in which sexualized deepfake abuse can be engaged in and experienced, and the overlap in victimization and perpetration.

Regarding the motivations underpinning sexualized deepfake abuse, the findings of our study indicate that theories of male peer support (DeKeseredy & Schwartz, 1993, 2013, 2016), techniques of neutralization (Sykes & Matza, 1957), and moral disengagement (Pina et al., 2021) can provide useful frameworks to help inform prevention responses. For our participants, peer reinforcement was most prevalent not in the context of a sexual conquest, but in relation to gaining status and reputation within a male peer group, primarily linked to positive reinforcement for, and acceptance of, their abusive behavior. This emerged from peers commenting on a perpetrator’s skillset in creating a realistic, but fabricated, sexualized deepfake image, or from humorous or other positive responses from sharing the image, even if part of the humor resonated with the potential harm the image could cause. The normalization of sexualizing another person without consent was also strongly present in the findings, with several male participants describing bonding over their sexualized deepfake abuse behaviors, suggesting a prioritizing of homosocial relations, which included objectifying women and same sex attracted men to reinforce their masculine status. In this regard, male peer support theory can be applied to understand how certain social dynamics among men can contribute to this form of abuse.

Our findings indicate that within certain male peer groups, creating and sharing sexualized deepfake imagery is both normalized and encouraged as a way to bond or gain status, and to assert further dominance and control in the reinforcement of traditional gender norms. This was evident in participants mentioning their male peers in responses to many different interview questions, such as those on motivations and when reflecting on the potential harms of sexualized deepfake abuse. This finding can be considered with respect to the operation of peer support in the reproduction of hegemonic masculinity norms that align men’s higher status with acts of dominance over perceived weak, subordinate, and/or “feminized” others (Connell, 1987; Connell & Messerschmidt, 2005). Our findings further support that men may feel pressured to participate in sexualized deepfake abuse behaviors to avoid being the subject of the imagery, or to deny injury, weakness, or victimization should they become the target of the behavior. Male peer support theory seeks to explain how men receive emotional and social support from their peers for engaging in abusive behaviors (DeKeseredy, 1988). This was evident in our study, whereby support for engaging in sexualized deepfake abuse was present through encouragement and validation. In this regard, it was evident that social and cultural factors can contribute to sexualized deepfake abuse behaviors, even if they are not directly targeted at women. These findings present a clear challenge in the prevention of sexualized deepfake abuse, as there are a range of factors that are required to disrupt the social dynamics that normalize and reinforce these harmful behaviors, starting with creating positive and respectful peer group cultures.

Techniques of neutralization and moral disengagement theory are also useful in helping understand how perpetrators in our study downplayed and minimized the harms of sexualized deepfake abuse (Sykes & Matza, 1957). Across the interviews, participants commonly denied responsibility, for example, claiming that AI technologies made it easy to create images, thus shifting blame away from themselves. Participants also denied injury to the victim, arguing that in their situations it was harmless or in some cases that the victim deserved it because they had previously caused the perpetrator harm, for example, ending a romantic relationship. This was also evident among victim responses who compared their experiences to those of physical violence. Our findings further support the use of appealing to higher loyalties as a neutralization technique for engaging in sexualized deepfake abuse, whereby perpetrators commonly justified their actions to fit in with their peer group, or in some cases, to retaliate against perceived wrongs. There was less evidence of perpetrators condemning the condemners, other than some perpetrators suggesting that labeling their behavior as abuse was being overly sensitive.

In relation to moral disengagement theory, perpetrators in our study were able to rationalize their actions using similar tactics to those identified by Pina et al. (2021), such as minimizing harm and blaming the victim. Further to this, there was evidence of perpetrators using euphemistic labeling, whereby they described their actions as “just having fun” and “sharing a joke” to downplay the potential severity of the abuse; advantageous comparison, comparing their actions to more severe forms of sexualized deepfake abuse where the intent was to harm; and diffusion and displacement of responsibility, blaming external factors such as peer pressure, and AI technologies. These mechanisms allowed perpetrators in our study to disengage from their moral standards, enabling them to reflect on their experiences engaging in sexualized deepfake abuse without expressing significant levels of guilt or responsibility.

Connected to the normalization of such behaviors is the accessibility and ease of creating non-consensual sexualized deepfake content. One of the more unique motivations and neutralization techniques to emerge—which is intimately linked to the accessibility of deepfake software, the ease with which images can be created, and social status building—was curiosity. While some research on sexting and non-consensual image sharing among young people has found curiosity to be a potentially motivating factor (Hollá, 2020; Yepez-Tito et al., 2021), this has primarily been sexual curiosity and experimentation, as opposed to our study, which found curiosity in the process of creating the fake image as a driving and facilitating factor in behaviors. This finding highlights another danger arising from sexualized deepfake abuse, also linked to actor-network theory (Latour, 2005), in that the convergence of human curiosity and seeking peer reinforcement, alongside the accessibility of tools and ease of creation, means essentially anyone could become a perpetrator, and anyone could become a victim of sexualized deepfake abuse. This is further connected with the absence of consequences for the behaviors. In our study, no perpetrator reported having been legally sanctioned for their behavior, and no victims had any legal action taken, even for the small number of victim participants who reported the abuse to police. Our findings suggest that the commercialization of AI tools, combined with the acceptance and normalization of creating non-consensual deepfake imagery and the perceived lack of punishment, has given a broader range of people who might otherwise not engage in sexualized deepfake abuse, the capacity, and motivation, to do so. The accessibility and ease with which sexualized deepfake abuse can be created from online interactions, or from lifting images from social media profiles, also suggests that these behaviors may become a dangerous tool for sextortion scams and perpetration, as reflected in the experiences of four victims in our study.

Accessibility and ease should not remove the onus from perpetrators. In our study, it was unusual for curiosity to be a sole motivation, and in every perpetrator story shared, the participant had engaged in both creating and then sharing the imagery, suggesting broader desires than curiosity. But it does highlight one of the dangers of having accessible, harmful AI tools, particularly when considering the broader social dynamics and norms arising from the male peer groups represented in our study. The unique ways in which peer reinforcement was expressed are particularly concerning, moving beyond sexual conquests and into creativity, or as Hall et al. (2021) similarly found in the context of upskirting perpetration, the “craftmanship” of the abusive image.

### Implications

Sexualized deepfake abuse shares similarities with other forms of TFSV, but it is distinct, even from other forms of IBSA. Comparing our findings with those of Flynn et al. (2022b), we identified more stranger and friend perpetration (as opposed to partner/ex-partner), and motivations that expanded beyond harm, control, and humor, to include monetary gain, peer reinforcement, and curiosity. There was also a clear pattern in minimizing the harms experienced, including by victims, which requires further exploration to ensure the potential consequences and impacts of sexualized deepfake abuse are not brushed aside due to the image being fake/altered (especially as technologies improve), or the perpetrator’s perceived motivation.

Understanding the normalization of sexualized deepfake abuse and the minimization of its harms through a lens of male peer support (DeKeseredy & Schwartz, 1993, 2013, 2016), techniques of neutralization (Sykes & Matza, 1957), and moral disengagement theory (Pina et al., 2021) is especially important for the creation of still-in-progress deterrence and criminalization efforts. This is particularly relevant given that the normalization of sexual violence affects legal processes, such as resulting in lesser charges and lighter sentencing for perpetrators (Temkin & Krahé, 2008). Understanding motivations, such as peer reinforcement, is also key in attempting to create prevention education through promoting respectful relationships. Existing prevention campaigns targeting IBSA and sexting more generally have been heavily critiqued for victim-blaming, for enforcing gender norms, and for ignoring the realities of contemporary sexual expression and intimacy (Albury & Crawford, 2012; Flynn et al., 2023a; Zauner, 2021). It is important that when developing awareness and education material on sexualized deepfake abuse that the motivations of curiosity, creativity, and peer reinforcement are appropriately engaged with to challenge gender roles and cultural and social norms that underpin the justification of harmful behaviors.

One of the most pressing findings relates to the accessibility and ease with which sexualized deepfake abuse can occur. Being able to locate tools through search engines or purchases of apps, as opposed to having to locate these tools on the dark web or illegal websites, contributes to its normalized use. As Flynn et al. (2024a) report, “nudify apps are readily accessible and advertised freely on people’s social media feeds, including Instagram and X. In Australia, a google search of ‘free deepnude apps’ brings up about 712,000 results.” While humans ultimately decide to engage in sexualized deepfake abuse, some disruption and prevention responses must be tailored toward the developers and hosts of these technologies to be answerable to community standards. As Brigham et al. (2024, p. 11) advocate, this could include “keyword-based warnings in search engines or advertisements that inform the viewer about the harms of consuming” sexualized deepfake abuse. It could also include requiring the removal of technologies or apps that promote or facilitate criminally harmful behaviors from platforms and websites. There are long-standing legal precedents in many countries for regulating deceitful, harmful expressions that others perceive as true, which could provide this framework (Flynn et al., 2024a). Similarly, there should be some responsibility put onto pornography websites in screening for deepfake distribution, particularly given that perpetrator and victim participants in our study said these were an obvious platform for sharing. If these websites supposedly only feature consensual adult content, then much deepfake content would be a violation.

Additional benefits would flow from tighter regulations of advertising tools and technologies that encourage people to engage in these criminally harmful behaviors. Limiting the availability of these tools and restricting their advertisement online may help reduce their accessibility and people’s engagement in these harmful behaviors. There is also the flow-on potential to limit some of the drift into engaging in sexualized deepfake abuse. Further research into who is being targeted with sexualized deepfake creation advertisements, where this is taking place, and the potential for new advertising regulations would provide a useful contribution to the field and future policy.

The criminalization of all forms of sexualized deepfake abuse is an important step toward recognizing its potential harms and increasing occurrence. IBSA laws exist in many countries, some of which already capture sexualized deepfake abuse (e.g., where there is specific reference to “created,” not just “taken,” or to “digitally altered images,” rather than just “nude or sexual images”; see, e.g., Flynn, 2023). There have been movements toward specifically criminalizing the non-consensual distribution of sexualized deepfake images; however, ambiguity in these laws remains as to whether the creation or the threat to create such content is also a crime. To the best of our knowledge, no laws currently criminalize the possession or seeking out of non-consensual sexualized deepfake content (of adults), which may contribute to downplaying the harms and normalizing the existence of such content. The law sends a powerful message that harmful behavior is not tolerated or condoned. While our findings suggest there is considerable crossover in perpetration between creating and then sharing the images, there is merit in governments exploring laws criminalizing the creation, threat, and possession of non-consensual sexualized deepfake imagery to reduce ambiguity and better recognize its potential harms, whether this involves reworking existing IBSA laws, or creating laws specific to AI-generated imagery.

### Limitations and Further Research

Like all research, this study has limitations. As a qualitative study with a non-probability, small sample size, the findings cannot claim to be considered representative of the Australian context or beyond. It is possible that because our study relied on self-reporting perpetrators and victims to describe their experiences that we did not capture a full representation of all forms of sexualized deepfake abuse. For example, in comparison to other qualitative research on IBSA, our study found only a small number of experiences involving partner/ex-partner contexts. We also recruited only two women identifying perpetrators and four men identifying victims, with no participants identifying as non-binary or transgender. Rather than this reflecting that sexualized deepfake abuse is not as common as IBSA in intimate partner contexts, that non-binary or transgender people do not experience or engage in sexualized deepfake abuse, or as evidence of the gendered characteristics of sexualized deepfake abuse, this should be considered a limitation of the recruitment strategy and/or research findings. The interviews were also conducted at a time when the Australian government was debating (and later introduced) legislation criminalizing the non-consensual sharing of sexualized deepfake imagery at the federal level (*Criminal Code Amendment [Deepfake Sexual Material] Bill 2024*). This legal change may have potentially impacted the willingness of people to discuss their behaviors. Despite the limitations, this is the first known study of sexualized deepfake abuse involving interviews with both perpetrators and victims, and it makes an important contribution to the small (but growing) body of research, by providing unique insights into perpetrator and victim experiences. It is now necessary for further research to replicate and extend this study. For example, additional qualitative research is required to explore the sexualized deepfake abuse experiences of underrepresented participants, including those identifying as non-binary or transgender. In addition, quantitative research is needed to examine if, and how, the insights gained from this study generalize to the broader population.

## Conclusion

This article has provided a unique study into sexualized deepfake abuse involving interviews with perpetrators and victims to identify patterns in perpetration, motivations, and how adapting existing theoretical explanations like male peer support (DeKeseredy & Schwartz, 1993, 2013, 2016), techniques of neutralization (Sykes & Matza, 1957), and moral disengagement (Pina et al., 2021) should be used to inform prevention responses. While sexualized deepfake abuse perpetration shares some parallels with other forms of TFSV, including IBSA, it is distinct in the accessibility and ease with which deepfakes can be created of *anyone* by *anyone*, largely without threat of perceived or actual consequences. In light of this, intervention and responses specific to this form of abuse, distinct from other forms of IBSA, such as cross-national collaborations to regulate deepfake tool availability, searches and advertisements, as well as education and awareness campaigns specific to the consumption of, and engagement in, sexualized deepfake abuse, are necessary as a starting point to tackle this emerging form of harm.

## Data Availability

Our ethical approval does not permit the qualitative data to be shared beyond the research team.
